# Testing the effectiveness and implementation of a brief version of the Common Elements Treatment Approach (CETA) in Ukraine: a study protocol for a randomized controlled trial

**DOI:** 10.1186/s13063-018-2752-y

**Published:** 2018-08-03

**Authors:** Laura K. Murray, Emily E. Haroz, S. Benjamin Doty, Namrita S. Singh, Sergey Bogdanov, Judith Bass, Shannon Dorsey, Paul Bolton

**Affiliations:** 1 Department of Mental Health, Johns Hopkins Bloomberg School of Public Health, 624 N. Broadway, Baltimore, MD 21205 USA; 2 Centre for Mental Health and Psychosocial Support, National University Kyiv-Mohyla Academy, Glasunova str 2/4, Kyiv, 01042 Ukraine; 3 Department of Psychology, University of Washington, Guthrie Hall (GTH), 119A 98195-1525, Seattle, WA 98105 USA; 4 Department of International Health, Johns Hopkins Bloomberg School of Public Health, 624 N. Wolfe Street, Baltimore, MD 21205 USA

**Keywords:** Global mental health, Transdiagnostic, Depression, Veterans, Internally displaced persons, Trauma, Cognitive-behavioral therapy, Implementation research

## Abstract

**Background:**

Mental illness is a major public health concern. Despite progress understanding which treatments work, a significant treatment gap remains. An ongoing concern is treatment length. Modular, flexible, transdiagnostic approaches have been offered as one solution to scalability challenges. The Common Elements Treatment Approach (CETA) is one such approach and offers the ability to treat a wide range of common mental health problems. CETA is supported by two randomized trials from low- and middle-income countries showing strong effectiveness and implementation outcomes.

**Methods/design:**

This trial evaluates the effectiveness and implementation of two versions of CETA using a non-inferiority design to test two primary hypotheses: (1) a brief five-session version of CETA (Brief CETA) will provide similar effectiveness for reducing the severity of common mental health problems such as depression, post-traumatic stress, impaired functioning, anxiety, and substance use problems compared with the standard 8–12-session version of CETA (Standard CETA); and (2) both Brief and Standard CETA will have superior impact on the outcomes compared to a wait-list control condition. For both hypotheses, the main effect will be assessed using longitudinal data and mixed-effects regression models over a 6-month period post baseline. A secondary aim includes exploration of implementation factors. Additional planned analyses will include exploration of: moderators of treatment impact by disorder severity and comorbidity; the impact of individual therapeutic components; and trends in symptom change between end of treatment and 6-month assessment for all participants.

**Discussion:**

This trial is the first rigorous study comparing a standard-length (8–12 sessions) modular, flexible, transdiagnostic, cognitive-behavioral approach to a shortened version of the approach (five sessions). Brief CETA entails “front-loading” with elements that research suggests are strong mechanisms of change. The study design will allow us to draw conclusions about the effects of both Brief and Standard CETA as well as which elements are integral to their mechanisms of action, informing future implementation and fidelity efforts. The results from this trial will inform future dissemination, implementation and scale-up of CETA in Ukraine and contribute to our understanding of the effects of modular, flexible, transdiagnostic approaches in similar contexts.

**Trial registration:**

ClinicalTrials.gov, ID: NCT03058302 (U.S. National Library of Medicine). Registered on 20 February 2017.

**Electronic supplementary material:**

The online version of this article (10.1186/s13063-018-2752-y) contains supplementary material, which is available to authorized users.

## Background

Mental, neurological, and substance use disorders account for 10.4% of the global burden of disease [1]. The field of global mental health has made significant advances over the past decade in understanding the effectiveness of various evidence-based mental health treatments in low- and middle-income countries (LMIC) [2–10]. Despite this progress, the availability of such evidence-based treatments is limited by multiple challenges around buy-in, scale-up, and sustainability. Research shows that most individuals with mental health needs do not receive care - creating a substantial mental health “treatment gap” [11–13].

One possible strategy for addressing the challenge of scale-up is utilizing a transdiagnostic approach, in which providers receive training in *one* approach that can address multiple common mental health problems and comorbidity [14]. The use of transdiagnostic approaches may prevent the need to train providers in multiple focal treatments, which creates challenges for scale-up in terms of time and cost, and has limited ability to address comorbidities. Various transdiagnostic models, some modular, initially were developed and tested in high-income settings, and have demonstrated effectiveness and provider appeal [15–18]. The term transdiagnostic is widely used with varying underlying conceptualizations. In their conceptualization, Sauer-Zavala and colleagues [19] describe three approaches: (1) a *universally applied principles approach*, which is based on a school of thought (e.g., psychodynamic, cognitive-behavioral, humanistic) that is then applied to multiple disorders regardless of symptom presentation; (2) a *modular or common-elements approach*, which involves using components or elements of existing evidence-based treatments that can be delivered in different orders and combinations to address symptom presentation and wide-ranging comorbidities; and (3) a *shared mechanism approach*, which is informed by theory and targets underlying processes of certain disorders. Other potential differences across transdiagnostic treatments, regardless of how they are conceptualized, include more structural aspects such as dosage or intensity (e.g., number of sessions), decomposability or modularity (the degree to which elements are mostly independent of one another), flexibility, linearity, and whether or not they are multi-problem [20].

Building on growing empirical support for transdiagnostic treatments and their potential promise for efficiency, our research team developed The Common Elements Treatment Approach (CETA) [21], a modular, flexible, transdiagnostic approach specifically for LMIC, in which task-sharing is used, and providers often have minimal or no formal mental health training. CETA was modeled on a treatment developed by Drs. John Weisz and Bruce Chorpita [18, 22–25], specifically to be a modular, flexible, transdiagnostic approach as that was believed to have the most potential to address implementation and scale-up barriers in LMIC. Such transdiagnostic models could address a wide range of comorbid problems across the life span (reducing the number of treatments needed), with the ability to only deliver the dosage needed (potentially reducing cost and resources). The goal in adapting an existing intervention—versus just using the existing intervention—was to create a more simplified approach that had a limited number of elements, simple language, a short manual with practical step-sheets, and decision rules giving providers and their supervisors flexible choices in element selection, sequencing, and dose. The decision rules help the provider use the client’s initial assessment (e.g., primary presenting problem; comorbidity) and ongoing presentation (e.g., symptom changes during treatment) to plan and adjust treatment as needed [21]. Our team has evaluated CETA effectiveness with adults in Iraq and Thailand, demonstrating that providers with minimal or no formal mental health training could provide CETA with fidelity and achieve positive outcomes with their clients (large effect sizes across outcomes) [2, 26].

Although the field of global mental health has a number of evidence-based mental health treatments with evidence of effectiveness, treatment duration is often cited as a significant barrier to scale-up and reduction of the treatment gap as the longer the treatment, the more staff and resources are needed, and the potential for dropout increases. Previous studies of cognitive-behavioral therapy (CBT)-based interventions, interpersonal psychotherapy, and CETA in LMIC have been tested with treatment durations of approximately 8–12 1 hour sessions [3, 7, 9, 26–28]. Scale-up challenges have led to a push to develop intervention models that are even briefer, particularly for highly mobile populations, such as trauma-affected groups or displaced persons [29]. A modular, flexible, transdiagnostic model provides an opportunity to decrease the length of treatment based on need (e.g., utilizing fewer elements), but still catering to the comorbid problems experienced by any population or individual.

There may be different methods to reducing the length of treatments. One approach to developing shorter interventions is to focus on elements or skills that may be easier to learn. Another approach is to identify treatment elements that seem to be essential, and, when treatments have been subjected to dismantling studies—account for greater variance in client outcomes. For example, although cognitive elements may be more challenging for lay providers and/or clients to understand and master, cognitive elements are also among those most frequently used in many evidence-based treatments for common mental health problems. Research suggests that cognitive interventions can be learned by lay counselors when provided with coaching and supervision [3, 9, 26, 28], and given their broad use in a number of evidence-based protocols may represent an important mechanism of action. Interventions using cognitive skills have been tested in LMIC and have shown evidence of effectiveness, often with large effect sizes. For example, in a study focused on survivors of sexual violence in Democratic Republic of Congo, Bass and colleagues [3] evaluated Cognitive Processing Therapy – Cognitive Therapy, which is an intervention that focuses primarily on cognitive elements. Effect sizes were greater than 1.0 for post-traumatic stress disorder (PTSD) and combined depression and anxiety. Trials in LMIC that used evidence-based treatments, including cognitive elements, with youth participants have also been shown to be effective, even when delivered by lay providers.

To explore whether the CETA approach could be provided in fewer sessions and with fewer elements, yet retain effectiveness, we designed a five-session intervention that is “front-loaded” with the elements that are most commonly used in evidence-based practices per distillation work [30], the empirical academic literature, and research studies conducted in LMIC with lay providers. Our working hypothesis is that these elements are the “strongest” mechanisms of action and, given past research in LMIC, are feasible elements for lay providers to deliver effectively. The study design is a non-inferiority trial and a trial of effectiveness by comparing a five-session, “front-loaded,” Brief version of CETA to Standard CETA (8–12 sessions) and both versions to a wait-list control.

## Methods/design

### Study aims

This trial is designed to test two primary study hypotheses: (1) that a brief five-session version of CETA (Brief CETA) will provide similar effectiveness for reducing the severity of depression and post-traumatic stress (PTS) symptoms and improving daily functioning compared with the standard 8–12-session version of CETA (Standard CETA) and (2) that both Brief and Standard CETA will provide substantial impact for these same outcomes compared to a wait-list control condition. For both hypotheses, the main effect will be assessed 6 months post baseline.

Secondary aims include an exploration of implementation factors, including treatment fidelity, barriers and facilitators to the intervention implementation at the client and provider levels, and an exploration of unexpected additional effects of the program, both positive and negative. Planned secondary analyses include exploration of moderators of treatment impact by severity of disorders and comorbidity, examination of the impact of individual therapeutic components, and trends in symptom change between end of treatment and 6-month assessment for those in either Brief or Standard CETA.

### Study context

This study is being conducted in Ukraine. Starting at the end of 2013 through early 2014, Ukraine experienced a period of rapidly increasing and widespread political discontent. In March 2014, Russia annexed Crimea in Eastern Ukraine, which began a series of violent events that continue today between pro-Russian separatists (supported by Russia) and Ukrainians supportive of the new government. These events have resulted in significant social and economic disruption and dislocation, with many individuals and families experiencing significant trauma and violence. This has included large numbers of individuals from across the country being conscripted into military service, hundreds of thousands of people being displaced from the eastern regions of Donetsk and Luhansk (the Donbass) and Crimea, and host communities being impacted by the influx of internally displaced persons. According to the Internal Displacement Monitoring Center [31], there are nearly 1.7 million internally displaced persons in Ukraine as of August 2015 with likely more who are displaced but not officially registered.

Between February and March of 2016, we conducted a brief qualitative study with internally displaced persons from the Donbass and military veterans living in two eastern oblasts, or regions, of Ukraine: Zaporizhia and Kharkiv. The aim of this formative study was to inform the adaptation of the CETA intervention to the Ukrainian context. The study sample included youth and adult internally displaced persons, veterans and their family members (wives and children), as well as community key informants and mental health professionals. We carried out free-list interviews, key informant interviews, and focus group discussions (FGDs) with a total of 226 participants. The qualitative study explored: participants’ perceptions of significant problems faced by internally displaced persons and veterans; descriptions of local functioning behaviors; and experiences with accessing, and delivering, mental health services.

Analysis allowed for the identification of psychosocial and mental health problems and symptoms of distress that were relevant to the study population. Both internally displaced persons and veterans described experiences of social isolation, difficulties with adaptation and (re-)integration, and conflicts with family members. Internally displaced persons also described missing home, fear, having a bad relationship with local residents/ lack of acceptance by the community, and conflicts in school among children. Veterans additionally named problems with substance use (alcohol and drug use), community members who are against them, feelings of being misunderstood by family members and the community, and aggression. For both groups, it was critical that mental health providers be trustworthy, accepting, and understanding of internally displaced persons and veterans’ status and experiences, as well as competent and effective.

### Study design

**Fig. 1:**
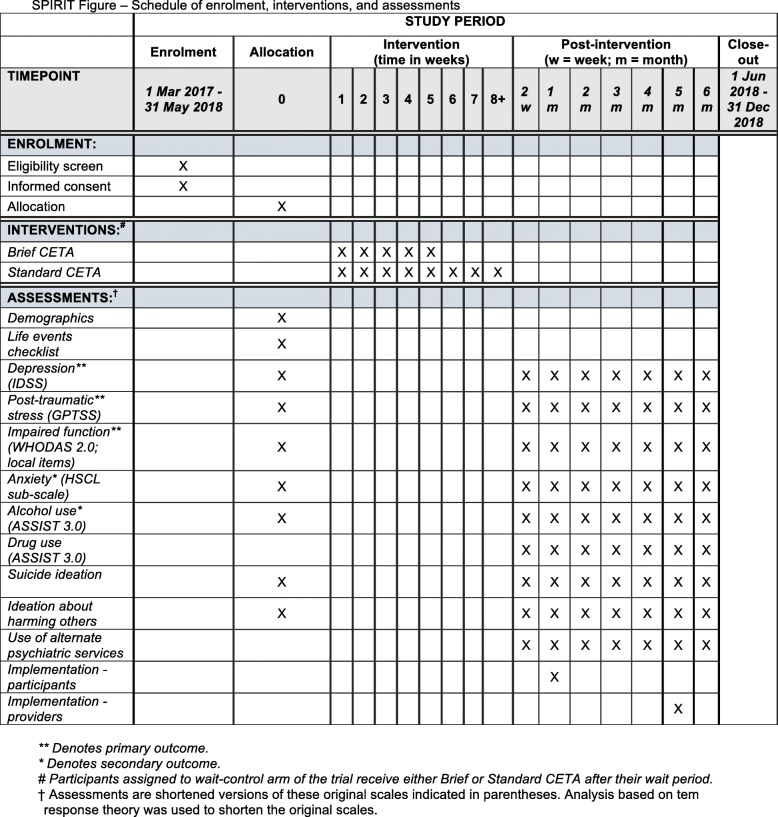
Standard Protocol Items: Recommendations for Interventional Trials (SPIRIT) Figure; schedule of enrollment, interventions, and assessments

This study is conducted as a Hybrid Implementation-Effectiveness Type II [32] three-armed, single-blinded, randomized controlled trial in three cities in Ukraine. The study compares Brief CETA to Standard CETA and both Brief and Standard CETA to a wait-list control condition on our primary outcomes of depression, PTS symptoms, and daily functioning. A secondary aim is to evaluate implementation outcomes. The study design adheres to the Standard Protocol Items: Recommendations for Intervention Trials (SPIRIT) guidelines, including a SPIRIT Flow diagram, SPIRIT Schedule, and Checklist (Fig. 1 and Additional file 1).

### Participants and eligibility criteria

Recruitment for this trial began in March 2017 and is ongoing. The first participant was enrolled in the trial on 8 March 2017. Participants in this trial are adults living in or near three Ukrainian cities—Kyiv, Kharkiv, and Zaporizhia—who meet the following inclusion criteria:

- Adults aged 18 years or older
- Being either an internally displaced person from Eastern Ukraine (the Donbass or Crimea); a veteran of the Anti-Terrorist Operations (ATO) in Ukraine (including adult family members); a volunteer in the “revolution of dignity” in the war in Eastern Ukraine, or in support of internally displaced persons or veterans from these conflicts; or any other non-military person affected by the conflict in Eastern Ukraine
- Elevated depression and/or PTS symptoms as defined by:
  - ○ A score of 7 or above on the locally validated depression scale (possible score range: 0–24) or
  - ○ A score of 9 or above on the locally validated Post-Traumatic Stress Scale (possible score range: 0–39) or
  - ○ A score of 7 or above on the locally validated depression scale *and* 9 or above on the locally validated Post-Traumatic Stress Scale
- Evidence of impaired daily functioning as assessed by a score of 4 or above on a locally validated scale of impaired functioning (possible score range: 0–32)
- Living in or around the three study cities for the duration of the study (at least 6 months) and able to regularly attend at least one of the locations where CETA is available

There are no restrictions on eligibility due to receiving other services. Participants who met the above criteria and who receive other health, mental health, social, behavioral, and any other type of services can be included.

Exclusion criteria include:

- An adult who is still a member of the armed forces (i.e., not a veteran)
- Acute suicidal or homicidal risk or active psychosis requiring immediate referral to psychiatric services
- Severe co-occurring medical problem that limits the ability to attend regular counseling sessions and/or requires immediate hospitalization

Assessors asked about suicide risk during the initial baseline assessment done on a tablet. If a possible participant responded “yes” to a single question about suicidal ideation in the past 2 weeks, they were asked four follow-up questions by the staff facilitating the baseline to gauge immediate risk. If someone answered positively to any of these follow-up questions, the assessor immediately called their supervisor, who provided clinical guidance by phone to assess for further risk, including past suicidal behavior. The supervisor was able to consult with clinical supervisors if they were unsure of the person’s level of risk. If the person was judged to be at immediate risk (i.e., past attempt and current ideation, a plan, and the means to carry out that plan) they were referred to a psychiatrist (who saw them within 24 hours), responded to by a local emergency psychiatric service, or driven directly to a hospital and were not randomized into a treatment condition. However, it was possible for a potential participant to be “cleared” by a psychiatrist and subsequently randomized into the trial. All clients were who were determined to have some risk, but not immediate risk, were asked to sign a safety contract and a safety plan was made to insure the person was checked on regularly and knew what to do and who to call if they started having suicidal thoughts. Any individual who could not respond to questions or presented with a severe illness was referred to a psychiatrist or physician and not included in the trial. Homicidal risk was not asked about explicitly, but if it came up during the baseline assessment or during any additional suicide safety procedures, the staff contacted a supervisor to create and sign a safety contract and made a referral to a psychiatrist to be seen within 24 hours.

### Recruitment and baseline assessment

**Fig. 2:**
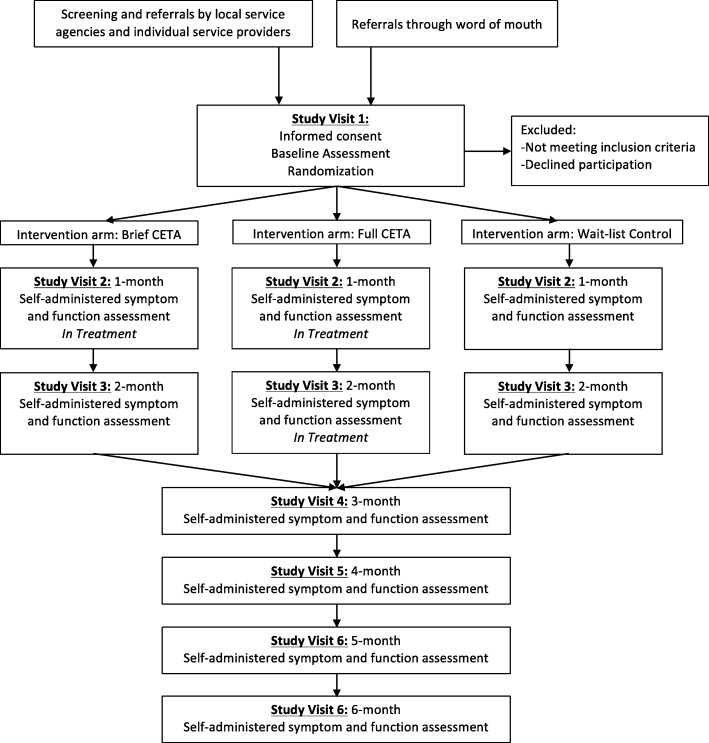
Study protocol flow

The participant flow is shown in Fig. 2. Both internally displaced persons and veterans are recruited through local service providers including government and non-governmental organizations (NGOs), through referral by study counselors from their existing client base, and through self-referral. Staff at the local NGOs were provided with a locally validated brief instrument that assessed symptoms of depression, anxiety, and PTS. This screening instrument is based on a previous validity study and Item Response Theory (IRT) analysis and includes the same symptom questions as the main study instrument, but does not ask about suicidal ideation or impaired functioning. As part of their regular services, the referring organizations use this instrument to assess their clientele for need of mental health services. When the instrument indicates depression and/or PTS symptoms, the person is informed about the study and asked if they agree to be contacted by a member of the study team.

Once a person has been identified as having elevated depression and/or PTS symptoms and agrees to be contacted, a study staff member sets a time to meet in person, complete consent procedures and conduct the baseline assessment to ensure trial eligibility. Both informed consent and the baseline assessment are self-administered on an electronic tablet. The study staff is present to facilitate the process, troubleshoot problems with the tablet and answer any questions, but will allow the potential participant to respond to questions in private. Consent is via a secure data collection/management software program (CommCare) [33] on a password-protected, handheld tablet. If the person does not consent to participate in the trial, the baseline assessment is not administered. If the person consents to the trial and completes the baseline, the person is immediately notified if they are eligible and whether they will receive treatment immediately or are asked to wait. If randomized to receive CETA, the participant is not told if they have been allocated to Brief or Standard CETA. Individuals who are not eligible for the study (either by not meeting the inclusion criteria or meeting one or more of the exclusion criteria) are thanked for their time and given a list of service organizations in their city.

#### Baseline assessment

The baseline assessment consists of demographic information and scales to measure our primary outcomes: symptoms of depression, PTS, and impaired functioning; and secondary outcomes: alcohol use and anxiety symptoms. Briefly, based on our initial qualitative work, we identified, adapted, and tested appropriate mental health scales, including the International Depression Symptom Scale (IDSS) [34], the Global Post-traumatic Stress Symptom Scale (GPTSS) [35], the Anxiety sub-scale of the Hopkins Symptom Checklist (HSCL-A), and the Alcohol Smoking and Substance Involvement Screening Test (ASSIST 3.0) [36, 37]. The IDSS and GPTSS were developed based on presentation of depression and PTS globally. For anxiety we used the HSCL-A [38] given its demonstrated validity in diverse populations, including those affected by political conflict [39–42]. For alcohol use, we used the alcohol section of the ASSIST 3.0, developed by the World Health Organization as a cross-cultural screener for substance use problems. It consists of eight questions, focused on frequency, dependence, and functional consequences of use. To assess impaired functioning (primary outcome), we used the World Health Organization’s Disability Adjustment Schedule 2.0 (WHODAS 2.0) 12-item version and a set of local function items. The WHODAS 2.0 is a generic instrument developed to provide a standardized measurement of functional impairment across cultures [43]. The set of local function items measures the degree of difficulty people experience when performing activities of daily living that are salient to the local population [44].

**Table 1:** Common Elements Treatment Approach (CETA) trial aim 1 evaluation measures

Measures	Description of measure	Number of items	Cronbach’s alpha	Test-retest reliability	Area under the curve^a^
Primary outcomes					
Depression symptoms	Items taken from the International Depression Symptom Scale (IDSS), a measurement instrument designed to represent global presentations of depression [27]	8	*α =* 0.89	*Rho =* 0.87	*AUC =* 0.78
Post-traumatic stress symptoms	Items taken from the Global Post Traumatic Stress Symptom Scale (GPTSSS) [28]	12	*α =* 0.91	*Rho =* 0.87	*AUC =* 0.68
Impaired function	A combination of items from the WHODAS [36] and a locally derived function scale based on qualitative data [37]	8	*α =* 0.88	*Rho =* 0.94	*AUC =* 0.72
Secondary outcomes					
Anxiety symptoms	Items taken from the Hopkins Symptom Checklist Anxiety sub-scale	4	*α =* 0.82	*Rho =* 0.80	*--* ^b^
Alcohol use	Items taken from the ASSIST alcohol sub-scale [29]	2	–	*Rho =* 0.80	*AUC =* 0.90

^a^When compared to corresponding diagnoses provided by Ukrainian psychiatrists using the Structured Clinical Interview for DSM-IV

^b^Insufficient sample size to calculate AUC due to only six participants diagnosed with generalized anxiety disorder

All of these outcome measures were translated into Russian and the full battery was tested during an instrument validation study among a sample of *N =* 153 internally displaced persons and veterans living in or around the urban areas of Zaporizhia and Kyiv [45]. The validation analyses involved testing reliability and validity, including criterion validity by comparing scores on the IDSS, GPSSS, HSCL-A, and ASSIST to local psychiatrist diagnosis using the Structured Clinical Interview for DSM-IV-Research Version (SCID-IV-RV) [46]. To further refine and shorten the measure, we performed a secondary data analysis of the validation data using IRT. The original assessment battery tested during the validation study had a total of 153 items, and IRT analysis resulted in a 37-item assessment that reliably and validly measures depression, PTS, impaired functioning, alcohol use and generalized anxiety (Table 1). Eligibility cutoff scores were developed by comparing average scores on each of the shortened measures to Structured Clinical Interview for DSM (SCID) diagnoses and generating scores that maximized both sensitivity and specificity for a given outcome [47]. These measures are administered at baseline for screening purposes and on a monthly basis and serve as the main study outcome (Table 2).

**Table 2:**
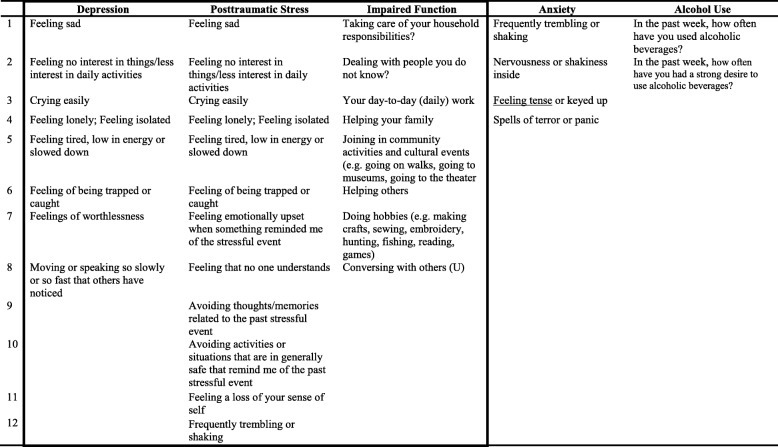
Items in each Common Elements Treatment Approach (CETA) trial aim 1 evaluation measure

*Outcomes in box indicate primary outcomes

### Study setting

The study is being conducted by researchers at the Johns Hopkins Bloomberg School of Public Health in collaboration with researchers from the National University of Kyiv-Mohyla University. The CETA treatment sessions take place at various locations in the three study sites in Ukraine—the urban areas of Kyiv, Kharkiv, and Zaporhizhia. Kyiv, the capital, is the largest city in Ukraine with a population of approximately 2.8 million [48] and is located in the north central part of the country. It has a large number of internally displaced persons and veterans. Kharkiv is the second largest city, with around 1.5 million residents, [48] and is located close to the Russian border in east Ukraine. It was the first capital city for Ukraine under the Soviet Union and is a prominent regional center of scientific and medical education. Zaporizhia has a population of around 800,000 is an important manufacturing and industrial hub in Southeastern Ukraine [48]. Neither Zaporizhia nor Kharkiv oblasts (regions) were occupied by Russian forces, but both border Donetsk and Luhansk and contain significant numbers of internally displaced persons and ATO veterans, as does Kyiv. Meeting locations vary, and are based on where participants feel most comfortable. Locations include private offices, veterans’ centers, internally displaced person service centers, and individual’s homes. A total of 39 Ukrainian psychologists, social workers, and veterans completed a training in CETA using the apprenticeship model [49]. Two authors (LKM, SD) were responsible for the training of the CETA providers and supervisors.

### Interventions

#### CETA intervention

As described in the introduction section, the Common Elements Treatment Approach (CETA) is a modular, flexible, transdiagnostic approach developed by our team specifically for use by providers who need not be formally trained mental health professionals. The number of elements was purposefully limited to help with training, mastery, and scalability. Elements are cognitive-behavioral and include: Engagement, Introduction, Thinking in a different way 1 and 2 (i.e., cognitive processing), Talking about difficult memories (i.e., imaginal exposure), Relaxation, Getting Active (i.e., Behavioral Activation), Problem solving, and a Cognitive-behavioral approach to substance abuse (see Table 3). The implementation of CETA elements can vary on: (1) selection of elements, (2) order of elements, and (3) dose of elements depending on symptom presentation. Providers are taught to use an assessment tool and what the client “does and says” to determine the primary problem(s). Elements are chosen based on the primary problem(s) with orders that mimic evidence-based treatments. For example, if someone comes in with primarily trauma-related problems such as nightmares, avoiding trauma-related stimuli, but also other problems (e.g., sadness, not engaging in pleasurable activities, relationship issues), an initial choice and order of elements would mimic trauma cognitive-behavioral treatments of: Engagement/Introduction, Thinking in a different way 1, Talking about difficult memories (usually two to five 1-hour sessions), and Thinking in a different way 2 (usually one to two 1-hour sessions). If depressive symptoms are still present after these elements are delivered, or if strong depressive symptoms are interfering, the element of Getting Active (i.e., Behavioral Activation) may be added. As developed, CETA can provide individualized targeting of treatment with the number of sessions delivered dependent on reduction in symptoms. Further details can be found in a description paper [21] and within the two clinical trials published to date on CETA [26, 28].

**Table 3:** Elements of Common Elements Treatment Approach (CETA)

Element	Simplified name (used in training)	Description
Psychoeducation and engagement	Introduction	• Focus on obstacles to engagement • Linking program to assisting with client’s problems • Includes family when appropriate. Program information (duration, content, expectations) • Normalization/validation of current symptoms/problems
Anxiety management strategies	Relaxation	• Strategies to improve physiological stress • Examples include: deep breathing, meditation, muscle relaxation, and imagery. Others added by local cultures
Behavioral Activation	Getting Active (GA)	• Identifying and engaging in pleasurable, mood-boosting, or efficacy-increasing activities
Cognitive coping/Restructuring	Thinking in a Different Way (TDW) – Part I and Part II TDW1 and TDW2	• Understand association between thoughts, feelings, and behavior • Learn to restructure thinking to be more accurate and/or helpful
Imaginal Gradual Exposure	Talking about trauma memories (TDM)	• Facing feared and avoided memories in detail • Gradual desensitization/exposure
Suicide/homicide/danger assessment and planning	Safety	• Assessing client risk for suicide, homicide, and domestic violence • Developing a focused plan with the client and client’s family (when appropriate) • Additional referral/reporting when needed
Cognitive-behavioral therapy (CBT) for substance use and relapse prevention	Substance use element (SU)	• Utilizes concepts of Motivational Interviewing to get client buy-in to change substance use/abuse behavior

**Fig. 3:**
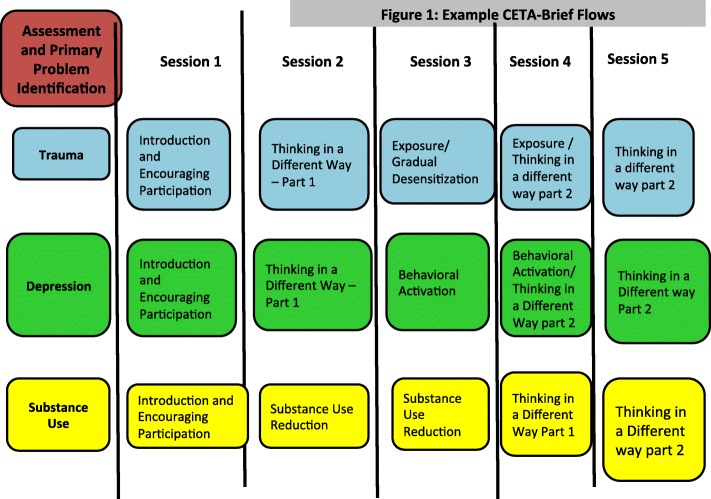
Example Common Elements Treatment Approach (CETA)-Brief flows

We developed a Brief CETA version with five sessions to address concerns around scalability and time limitations within low-resource settings and certain contexts. All Brief CETA flows include one cognitive element and one behavioral element, and an introduction session. (See Fig. 3). Counselors, supervisors and trainers first agree on a primary presenting problem (trauma, depression, anxiety, or substance use). Each problem area has a set flow of certain elements based on evidence-based treatments for these primary areas. For example, the two most commonly used evidence-based elements in treatments for trauma are Cognitive coping/restructuring and Gradual desensitization or exposure [50, 51]. For depression, most evidence-based treatments include Cognitive coping/Restructuring and/or Behavioral Activation, thus the Brief CETA flow contains only these. Since Cognitive coping/Restructuring is present in all flows, this may be used to target multiple or varying symptomatology (e.g., depression thoughts, unhelpful thoughts related to the traumatic event).

Since randomization occurs after the fourth session, all participants begin with one of the Brief CETA five-session flows. Those randomized to Standard CETA receive additional sessions based on the Standard CETA protocol including evaluating: (1) client report of symptoms, (2) what the client does and says, and (3) supervision. A provider needs to provide at least three additional sessions, and more could be provided as needed. Completion of Standard CETA is based on decreased symptoms, improvement report by client, and supervisor approval. This Standard CETA arm models a clinical “stepped care” approach wherein a client would be offered a “front-loaded Brief CETA” first, and then if symptoms persist, treatment can be augmented with additional sessions and/or elements [52].

### Control condition

Participants assigned to the wait-list control condition are asked to wait for approximately 6 months before being offered the Stanard CETA intervention. Wait-list control participants undergo monthly monitoring after enrollment in the study. Monitoring is done via in-person meeting or, if necessary, a phone call, to ensure ongoing connection with the study; to obtain information on current mental health status including high-risk symptoms (i.e., suicidality) that would require immediate assistance; and to record any other services they receive. Control participants are administered the same symptom items and impaired function scale as the baseline assessment as CETA participants. Study monitoring and evaluation staff are responsible for these monthly assessments. During the wait period, participants are free to use other social and mental health services. A single item on the assessment asks whether the participant has obtained other services, and if so, which ones. Study monitoring and evaluation staff provides participants with a list of other psychosocial services in their respective cities during the baseline assessment visit.

### CETA providers and training

Thirty-nine Ukrainian individuals were initially trained, with 34 continuing onto the study (seven men; 32 women). They hold a range of positions including social worker (9), psychologist (12), volunteers (e.g., with psychological crisis services) (5), physician (1), program manager (4), teacher/lecturer (2), and lawyer (1). A psychologist in Ukraine will have received approximately 2 years of post-university training in psychological theory but without applied clinical experience. Social workers are usually based within social services and are not trained to provide psychological treatments. In the training we found that most trainees did not have experience providing talk therapy directly to clients. Most were educated in Gestalt Theory and had little background in CBT.

CETA training and supervision followed the Apprenticeship Model (see Murray et al. [49] for details). Ukrainian providers (*N* = 39) received a 10-day training in CETA (May 2016), and then subsequently participated in small practice groups led by local supervisors (July 2016 to February 2017). They were taught all components, as well as how to make decisions about selection, sequencing, and dosing (i.e., tailoring to the individual participant) based on three sources of information: (1) results from certain items on the validated study instrument, (2) client observations and statements in the assessment and early sessions, and (3) discussion with their supervisor.

Initially, eight supervisors were included in supervision training. Some were not able to commit to the time required for supervision, leaving five supervisors for the trial (four women). Two are psychologists, one of whom is also a professor of psychology at the National University of Kyiv-Mohyla Academy. One is a social worker, one is a project coordinator, and another is a supervisor for a substance abuse foundation program. The supervisors received an additional 8 hours during the initial training to review supervision skills, although at least 4 hours of this time ended up being logistical problem solving. From April 2016, supervisors are in weekly communication with a CETA trainer to review the agendas, cases, implementation of elements, decision-making on CETA, weekly symptom monitoring scores, what happens each session, and supervision skills [21]. These calls are usually through Skype and last approximately 2 hours each.

### Intervention fidelity

Fidelity to CETA is tracked through a three-tiered system. First, CETA providers complete session notes and report to their supervisors each week, providing self-report of fidelity. These session notes include: date, session number, elements done in session and for what duration, weekly monitoring scores, “yes/no” to suicide/homicide risk questions, details about what was actually done in session (qualitatively), and the plan for the next session. Second, informed by this information and additional queries and discussion between the counselor and supervisor (e.g., “tell me exactly how you introduced the thinking in a different way triangle to your client.”), supervisors document this same information in an Excel sheet. Supervisors also complete their own ratings evaluating providers’ fidelity, including: (1) adherence (i.e., how well the counselor followed the elements step-sheets), (2) review of homework, (3) engagement, (4) explaining the what and why of the element used, (5) competency (i.e., overall skill at teaching the element), (6) assigning homework, and (7) an overall, global rating of the counselor for that session. Ratings for each fidelity domain are done on a 1–5-point scale with 1 being the lowest rating and 5 being the highest rating. Third, each week the trainer reviews the local supervisors’ spreadsheet documentation and ratings (via a shared Excel file). Trainers document the time supervisors spent in supervision, what was covered, and the time spent between a supervisor and trainer. The trainer then rates the supervisors’ ability on: (1) ability to notice mistakes, (2) responding to counselor questions or mistakes with correct direction, (3) ability to objectively report to the trainer, (4) the number of counselors who need to change the plan per the trainer, and (5) an overall rating. If a counselor did not adhere to the plan or did not implement the element following the steps, they repeat the element or the steps they missed in the following session. No counselor is allowed to have a session until they have discussed the previous session with the supervisor, and the supervisor has discussed it with the trainer. Any safety concerns (e.g., suicidal) are documented in a safety log, and reported immediately to the CETA trainers and study investigators. Each case is addressed individually based on clinical needs and may include daily safety checks, safety contracts, and possibly referral to a psychiatrist. Fidelity documentation also tracks any changes to the flows throughout treatment, why the change was made, and by whom. This will allow us to look at the correlation and learning time of decision-making.

### Outcome and study measures

The study’s primary aim is to determine intervention effectiveness comparing Standard to Brief CETA, and both to a control group. For this aim, the primary outcomes are symptoms of depression, PTS, and impaired functioning (Tables 1 and 2) measured on a monthly basis for 6 months. Secondary outcomes include symptoms of anxiety and problematic alcohol use, also measured on a monthly basis for 6 months (see Tables 1 and 2 and Fig. 1). Outcome assessments for our primary aim of determining intervention effectiveness are done at baseline prior to randomization, and on a monthly basis for approximately 6 months.

All data is collected via self-administered digital survey on an electronic tablet device using CommCare [33]. Baseline assessments are done at a central location or at another private location convenient to the participants. Monthly assessments consist of the same symptom and functioning items as the baseline assessment (Table 2). For the control group, the study staff sets up monthly assessment appointments to be done at a central location and are self-administered on CommCare with the same symptom and functioning items as the baseline assessment (Table 2).

For the treatment groups, the monthly assessment is incorporated into a clinical monitoring form (Table 2). The clinical monitoring form includes the impaired function items every fourth visit (i.e., monthly) rather than on a weekly basis. The clinical monitoring form is self-administered on CommCare at the beginning of each CETA session and helps guide the course of treatment. After treatment ends, participants continue to be monitored monthly with the same assessment (Table 2) via in-person visit (or phone call if in-person is not possible) by the study team. At the last assessment (6 months post treatment), some additional demographic- and alcohol-related questions are administered to measure potential pre-post change on these characteristics.

A second aim of the study is to explore the implementation of CETA from the perspective of consumers and providers in the Ukrainian context, using a Hybrid Type II effectiveness-implementation design. To measure implementation factors, including barriers and facilitators to implementation, we use scales that cover the major implementation science domains of Adoption, Acceptability, Appropriateness, Feasibility, and Accessibility/Reach. These scales were previously tested among a sample of Ukrainian consumers and providers and found to be reliable and valid (Haroz et al: Measuring implementation in global mental health: an example from eastern Ukraine, under review). For consumers, these scales are administered via CommCare to Brief and Standard CETA participants at their second post-treatment monitoring assessment. Qualitative interviews with CETA participants (*N =* 60) are also conducted at this time. The qualitative interviews, using free listing, ask CETA participants about changes that they experienced during treatment, because of treatment, and what they would want to change about CETA. For CETA providers (counselors and supervisors), the implementation scales include additional constructs related to organizational leadership and capacity for sustainability, and will be administered near the end of the trial. Providers also participate in focus groups after 6 months of providing CETA services in order to discuss what they like, do not like, and what they would change about implementation of CETA.

### Randomization, concealment, un-blinding

All eligible and consented adults are randomly allocated by a computer-generated randomization algorithm to either a wait-list control condition, the Brief CETA model, or the Standard CETA model. The unit of randomization is the individual. Block randomization is used to ensure equal distribution of treatment and control participants across providers. Within a provider, we randomly allocate 20 participants using a 1:2:2 (control:Brief:Standard) allocation ratio. Participants, counselors, study staff, and all clinical supervisors are blinded to study group allocation between Brief and Standard CETA conditions. This is done in an effort to ensure that all participants across both Brief and Standard CETA conditions will receive comparable treatment (see section “Concealment” below).

Study group allocation, either treatment or control only, is revealed to eligible participants immediately following the baseline assessment. If assigned to begin treatment right away, the study staff assigns a participant to a counselor based on availability and location of treatment. Counselors set up an initial meeting with the participant within 10 days of the baseline assessment. If assigned to control, the study staff set up a study assessment visit approximately 30 days after the completed baseline assessment.

#### Concealment

At the initiation of treatment, all CETA providers, supervisors and trainers and participants are blind to whether a participant is randomized to Brief or Standard CETA. The informed consent describes the possibility of Brief vs. Standard to the participant. At the end of the fourth treatment session, the counselor reads a script to the participant reminding them that the next session may be the last session. Following completion of the fourth treatment session, the study staff team notifies the Johns Hopkins University (JHU) research team via email about the need to un-blind the Brief or Standard status. Only the study director and two analysts on the data monitoring team have access to the password-protected database linking study ID number to treatment status. Once an email request for un-blinding is received, the study director checks the randomized assignment and sends an email back to the research team, the supervisor, and the trainer with the participant status (Brief or Standard). The clinical supervisor then contacts the counselor to un-blind. This whole process is done within 24 hours of completion of the fourth treatment session so as to allow planning between counselor and supervisor for either ending or continuing treatment. The participant does not find out about Brief vs. Standard status until they arrive at the fifth session. If the participant is assigned to Brief but still interested in services, the counselor may give them a list of local services but may not continue seeing the client. The client is also prohibited from receiving CETA from another counselor while the trial is ongoing.

### Sample size

All sample size and power calculations are based on longitudinal data collected monthly for controls and at every fourth visit for the CETA clients groups over the course of 6 months. (seven measurement time points). For our primary hypothesis, that Brief and Standard CETA would result in similar changes in symptoms of depression, PTS and impaired functioning, we used a standard deviation (SD) of 7.0 for depression symptoms at baseline (based on previous studies among trauma-affected populations) [26, 28]. Under the assumption that differences in outcomes that are less than one third of the standard deviation are considered not practically meaningful [53, 54], a non-inferiority margin of *Δ <* 3 was selected. The sample size that would yield a power of 0.80 (*β* = 0.20) or greater in testing at *α* = 0.05 level of significance, with an assumed SD of 7.0 and a non-inferiority limit of *d =* 2.4 is *N =* 178 (or *n =* 89 in each CETA group). For aim 2, the sample size that would yield a power of 0.80 (*β* = 0.20) or greater in testing at *α* = 0.025 level of significance (adjusted for multiple comparisons), to detect a medium effect (*f =* 0.25) for the direct comparison of either Brief or Standard CETA to wait-list control would be *n =* 45 in each group. As the sample size needed for aim 1 is the larger of the two calculations, we will use it as the basis for the total study sample planning (*n* = 223 across the three arms; 89 in Brief CETA; 89 in Standard CETA; 45 in the control group). Accounting for 30% attrition, a total of *N =* 294 participants are needed for the randomised controlled trial (RCT).

For the implementation portion of the study, all study participants in both CETA arms are interviewed using a quantitative instrument (Consumer dissemination and implementation (D&I) Instrument) but only the first 30 completers in each CETA arm receive the qualitative interview. All CETA providers (approximately 40) are interviewed with a quantitative instrument (Provider D&I Instrument) and asked to participate in the FGDs.

### Data analyses

#### Primary analyses

Study hypotheses will be tested using an “intent-to-treat” model. The primary and secondary outcomes of interest will be mental health symptoms and functioning measured monthly for 6 months for all groups. Given the longitudinal nature of the data, we will use mixed-effects regression models to estimate average treatment effects while accounting for shared variance of repeated measures within participant. Treatment effects will be measured based on within-person change in mean scores on the symptom and function measures. Statistical significance will be set at *p <* 0.05. We will use Cohen’s *d* effect sizes to express the magnitude of the difference between treatment groups. Missing data will be explored as to the mechanism of missingness. If appropriate to assume missing completely at random or missing at random, we will use multiple imputation with chained equations to impute all missing data, including outcomes for those lost to follow-up [55, 56].

##### Analysis for secondary aims

Analysis of the implementation data will include examination of distribution of scale-level scores represented as average responses for all items in each scale. Individual-level items with average scores lower than the average score on the scale will be considered possible barriers and used to identify program implementation areas that could be improved. Finally, we will examine whether more positive experiences of implementation, as measured by higher average scores on each implementation scale, is associated with different treatment conditions, client outcomes, and provider characteristics. For the free listing responses from consumers, a full list of these responses and the frequency with which they were reported will be compiled. The provider FGDs will be analyzed using methods described in the Design, Implementation, Monitoring and Evaluation (DIME) manual 1 [57]. Briefly, notes will be taken during discussions and then thematically coded using emergent coding. Notes and coding will be done in the local language and then summarized before translation.

#### Planned secondary analyses

We will examine changes in client symptom trajectories after delivery of each component of CETA (i.e., elevation, slope, and both elevation and slope), using the clinical monitoring form (symptom questions only in Table 2). To do this, we will use the modeling approach detailed by Singer and Willet [58] to examine changes in client symptom trajectories after delivery of each component of CETA (i.e., elevation, slope, and both elevation and slope). This approach involves adding time-varying dichotomous indicators representing whether each participant has completed each of CETA’s components. This will allow us to look at whether clients’ symptom levels significantly decreased (elevation) or whether they had significantly faster improvement (slope) immediately following completion of certain components. We will compare these changes in trajectories to monthly change in the control group participants.

We will further analyze the clinical monitoring from data (symptom questions only in Table 2) by using survival analysis to calculate the number of sessions that it takes for clients to achieve reliable change using the Reliable Change Index (RCI) [59]. Clients who end or drop out of treatment will be censored. We will use control group data to predict the number of sessions required to achieve improvement in the absence of intervention. We will then adjust for the difference between the average improvement slopes for each condition in the survival analysis.

### Data management and dissemination plan

All data is collected via password-protected tablet using the CommCare mobile data collection service. Data is stored in a secure web-based portal. The data management team maintains a log of known data errors. Each week the data analyst exports the data to track eligibility and enrollment numbers and report to the larger study team. The investigators of the study will not perform any interim analysis. The primary outcome is the 6-month follow-up, so no impact analyses will be conducted prior to the completion of the trial.

Upon completion of the trial, analysis will be completed based on the aforementioned analysis plan using the full intent-to-treat sample. Results will be summarized for a final report to the funder and disseminated during meetings with Ukrainian government and NGOs, as well as, international NGOs who are working in the region. A plain language summary will be offered to community members and throughout public domains. Data will be stored on an encrypted drive and access will be granted to investigators who are added to the IRB as appropriate. De-identified data will be made available on request.

## Discussion

This trial uses a robust design that allows us to compare the effectiveness of two interventions using a non-inferiority design. To our knowledge, this is the first trial evaluating a five-session modular, flexible, transdiagnostic treatment. We remained consistent with the cognitive-behavioral focus of this version of CETA by offering at least one cognitive and one behavioral element in each flow. The National Institute for Health and Care Excellence (NICE) guidelines have cognitive-behavioral therapy as one of the top recommendations for the effective treatment of depression, PTSD, and substance use disorders [60].

Rather than focus on elements that may be easier to teach, our approach was to “front-load” CETA with elements that appear to be strong mechanisms of action. Cognitive coping and restructuring is a key element in most evidence-based treatments for trauma, depression, anxiety and substance use [9, 24, 50, 51]. Although often seen as a more “challenging” element, this cognitive skill is regularly taught to children and even preschoolers effectively [61] and has been used by lay providers with fidelity [3, 9, 26, 28, 62]. Exposure is a primary element in both PTS and anxiety disorders and is one of the most extensively used and studied elements in CBT [63]. This is often an element that providers may be anxious about implementing, and some providers initially believe it may be harmful [64] despite extensive evidence to the contrary [65–68]. We do recognize that these elements are likely to take longer for a provider to master compared to elements like Relaxation or Problem solving. Our rationale is that to obtain maximum symptom relief in a shorter time period, it may be necessary to have the strongest elements present.

This will also be the first trial directly comparing a brief versus standard course of CETA. While CETA demonstrated strong effectiveness on trauma, depression, and anxiety symptoms in the two completed trials [26, 28], 8–12 sessions can be lengthy for some clients. An important empirical question is the level of effectiveness that can be obtained with shorter durations of treatment and practice of CBT skills, and whether this varies by type of presenting problems or client. This has important implications for further scale-up and sustainability of community-based psychotherapy services; namely, 'can we effectively reach more people faster, with potentially lower costs, to reduce population morbidity of common mental health problems?

The implementation of CETA in this study is different from the two completed trials in that we allow counselors more decision-making flexibility. For example, counselors are able to choose a primary problem of substance use, anxiety, depression or trauma whereas in the two completed trials all started with a trauma flow based on more restricted inclusion criteria. For those participants who are randomized to Standard CETA, providers have full flexibility in choosing the elements, order, and dosing that they provide after the first five sessions. We are also tracking the creation of flows by counselors, supervisors, and trainers separately to better understand the amount of training needed for providers to master clinical decision-making about client need and match with an element(s).

Our planned secondary analysis will explore how and when people recover by examining the impact of individual elements and length of time in treatment. Results from this study design will allow us to draw conclusions about the effects of both Brief and Standard CETA as well as about which elements are integral mechanisms of action, informing future implementation and fidelity efforts. In addition, our exploration of the implementation of CETA from the perspective of consumers and providers in the Ukrainian context, using a mixed-methods approach will inform the literature on Adoption, Acceptability, Appropriateness, Feasibility, and Accessibility/Reach of CETA. The quantitative measures being used will add to further refinement of implementation science measurement for low- and middle-income contexts.

A brief modular, flexible, transdiagnostic approach is particularly useful when working in settings with displaced and trauma-affected populations, given the high rates of comorbidity and high mobility in some settings. The results from this trial will inform future dissemination, implementation and scale-up of CETA in Ukraine as well as contribute to our understanding of these approaches in similar LMIC contexts.

## Trial status

The trial is registered at ClinicalTrials.gov (NCT03058302; date of registration: 20 February 2017).

Recruitment began on 8 March 2017 and is expected to end on 1 June 2018.

## Additional file

Additional file 1: Standard Protocol Items: Recommendations for Interventional Trials (SPIRIT) 2013 Checklist: recommended items to address in a clinical trial protocol and related documents*. (DOC 121 kb)

## Abbreviations

ASSIST: Alcohol Smoking and Substance Involvement Screening Test
CBT: Cognitive-behavioral therapy
CETA: Common Elements Treatment Approach
D&I: Dissemination and implementation
GPTSS: Global Post-traumatic Symptom Scale
HSCL-A: Hopkins Symptom Checklist-Anxiety sub-scale
IDSS: International Depression Screening Scale
JHU: Johns Hopkins University
LMIC: Low- and middle-income countries
NGO: Non-governmental organization
NICE: National Institute for Health and Care Excellence
PTS: Post-traumatic stress
PTSD: Post-traumatic Stress Disorder
RCI: Reliable Change Index
SCID: Structured Clinical Interview for DSM
SCID-IV-RV: Structured Clinical Interview for DSM-IV-Research Version
SPIRIT: Standard Protocol Items: Recommendations for Intervention Trials
USAID: United States Agency for International Development
WHODAS: World Health Organization Disability Adjustment Schedule
